# Antioxidants Improve the Phenotypes of Dilated Cardiomyopathy and Muscle Fatigue in Mitochondrial Superoxide Dismutase-Deficient Mice

**DOI:** 10.3390/molecules18021383

**Published:** 2013-01-24

**Authors:** Hirofumi Koyama, Hidetoshi Nojiri, Satoru Kawakami, Tadahiro Sunagawa, Takuji Shirasawa, Takahiko Shimizu

**Affiliations:** 1 Department of Advanced Aging Medicine, Chiba University Graduate School of Medicine, Chiba 260-8670, Japan; 2 Department of Orthopaedics, Juntendo University School of Medicine, Bunkyo-ku, Tokyo 113-8421, Japan; 3 Molecular Gerontology, Tokyo Metropolitan Institute of Gerontology, Itabashi-ku, Tokyo 173-0015, Japan; 4 Research Laboratories for Applied Technology of Food, Asahi Group Holdings, Ltd., Ibaraki 302-0106, Japan; 5 Department of Ageing Control Medicine, Juntendo University Graduate School of Medicine, Bunkyo-ku, Tokyo 113-0033, Japan

**Keywords:** manganese superoxide dismutase (Mn-SOD), mitochondria, dilated cardiomyopathy (DCM), EUK-8, procyanidins

## Abstract

Redox imbalance elevates the reactive oxygen species (ROS) level in cells and promotes age-related diseases. Superoxide dismutases (SODs) are antioxidative enzymes that catalyze the degradation of ROS. There are three SOD isoforms: SOD1/CuZn-SOD, SOD2/Mn-SOD, and SOD3/EC-SOD. SOD2, which is localized in the mitochondria, is an essential enzyme required for mouse survival, and systemic knockout causes neonatal lethality in mice. To investigate the physiological function of SOD2 in adult mice, we generated a conditional *Sod2* knockout mouse using a Cre-loxP system. When *Sod2* was specifically deleted in the heart and muscle, all mice exhibited dilated cardiomyopathy (DCM) and died by six months of age. On the other hand, when *Sod2* was specifically deleted in the skeletal muscle, mice showed severe exercise disturbance without morphological abnormalities. These provide useful model of DCM and muscle fatigue. In this review, we summarize the impact of antioxidants, which were able to regulate mitochondrial superoxide generation and improve the phenotypes of the DCM and the muscle fatigue in mice.

## 1. Introduction

An imbalance between the oxidation by reactive oxygen species (ROS) and reduction by antioxidant systems induces intracellular oxidative stress leading to the initiation and progression of age-related diseases, including diabetes, hypertension, atherosclerosis, osteoporosis, and neuro-degenerative diseases. ROS include several harmful species, such as superoxide anion (O_2_^•−^), hydrogen peroxide (H_2_O_2_), and the hydroxyl radical (HO^−^). They are physiologically generated by mitochondrial respiration, as well as cellular enzymatic reactions in response to environmental stimuli. In antioxidant systems, enzymes (dismutase, catalase, and peroxidase) and small molecules (glutathione and vitamins *etc.*) finally detoxify ROS to non-toxic metabolites, such as molecular oxygen and water [1]. Superoxide dismutases (SODs) are the main antioxidant enzymes that catalyze the conversion of superoxide anion (O_2_^•−^ to hydrogen peroxide (H_2_O_2_) and protect cells and tissues from the reactive oxygen species (ROS) generated from endogenous and exogenous sources. Three SOD isoforms are expressed in mammalian cells: copper/zinc SOD (CuZn-SOD, SOD1), which is located in the cytoplasm [2]; manganese SOD (Mn-SOD, SOD2), which is localized in the mitochondrial matrix [3]; and extracellular SOD (EC-SOD, SOD3) [4]. A small fraction of CuZn-SOD has also been reported to be present in the intermembrane space of the mitochondria [5]. Mitochondria are both a major source of ROS production from the respiratory chain and a major target of ROS-induced cellular injury [6]. Therefore, mitochondrial Mn-SOD is thought to play an important role in the cellular defense against oxidative damage by ROS.

The pathological consequences of increased mitochondrial ROS production have been thoroughly analyzed in model animals, such as Mn-SOD-knockout mice. These mice die from severe dilated cardiomyopathy within several weeks of birth and exhibit striking lipid deposits in the liver, as well as neurodegeneration [7,8,9]. The mice also show a significant reduction in the activity of mitochodrial respiratory enzymes, including complexes I, II, and III and the TCA cycle enzyme, aconitase [10]. Furthermore, genomic DNA prepared from the hearts and the brains of Mn-SOD-deficient mice has been reported to show a significant accumulation of oxidative DNA damage [10]. Since Mn-SOD-deficient mice die within several weeks of birth, it is not possible to investigate the pathological consequences of oxidative damage in adult tissues or the physiological aging process *in vivo*. We have also argued that the phenotypes of such mice are too complex to obtain information about specific aging processes for each tissue *in vivo*. In order to devise a mouse model in which the Mn-SOD deficiency could be directed to specific tissues, we designed a conditional knockout allele using the Cre-loxP system.

## 2. Heart and Muscle-Specific Mn-SOD-Deficient Mice

**Figure 1:**
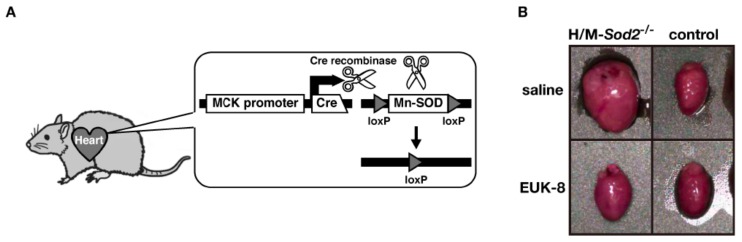
Heart and muscle-specific Mn-SOD-deficient mice. (**A**) Cre recombinase is expressed under the control of a heart and muscle-specific muscle creatine kinase (MCK) promoter. Cre recombinase recombines the loxP sites selectively to generate heart and muscle-specific Mn-SOD-deficient mice; (**B**) Isolated hearts from a H/M-*Sod2*^−/−^ mouse (top left) and a littermate control mouse (top right) at eight weeks of age. EUK-8 treatment improved the enlarged heart of a H/M-*Sod2*^−/−^ mouse (bottom left).

Oxidative stress plays a role in the development of heart failure [11,12]. To investigate the pathological role of mitochondrial ROS in the heart and muscle, we generated conditional Mn-SOD knockout mice by targeting these tissues (Figure 1A) [13]. We used muscle creatine kinase (MCK)-Cre transgenic mice to provide selective expression of the Cre protein in muscle tissues [14]. The Western blot analyses confirmed the specific loss of Mn-SOD expression in the heart and skeletal muscle of the heart and muscle-specific Mn-SOD-deficient mice (H/M-*Sod2*^−/−^ mice), but no loss was observed in the control mice. In the neonatal stage, we were unable to find any differences in the appearance or body size between H/M-*Sod2*^−/−^ and control mice. However, at two months of age, the H/M-*Sod2*^−/−^ mice began to exhibit growth retardation. At four months of age, the H/M-*Sod2*^−/−^ mice showed a 25% reduction in body weight compared to control mice, without any distinct muscle atrophy. By six months of age, all H/M-*Sod2*^−/−^ mutant mice had died (the median survival time was about four months). When examined macroscopically, all of the hearts from the H/M-*Sod2*^−/−^ mice showed cardiac enlargement at four months of age (Figure 1B). Transverse sections of the hearts from the H/M-*Sod2*^−/−^ mice showed a marked dilation of both the left and right ventricles, which was compatible with the end stage of dilated cardiomyopathy. In histological sections with Azan staining, diffuse fibrotic scars surrounded the myocardial cells. Some of the thickened fibrotic foci were due to necrotic changes in the myocardium. These results indicate that the H/M-*Sod2*^−/−^ mice progressively develop dilated cardiomyopathy.

To understand the biochemical alterations involved in the pathogenesis of cardiomyopathy, we examined the mitochondrial respiratory functions in the hearts of H/M-*Sod2*^−/−^ mice. Using an enzymatic histochemical analysis, we observed that there was a specific loss of succinate dehydrogenase (SDH, Complex II) activity, but not cytochrome c oxidase (COX, Complex IV) in the mutant hearts [15]. Among the mitochondrial respiratory chain complexes, SDH is particularly vulnerable to O_2_^•−^ because of its Fe-S center. Enhanced O_2_^•−^ generation due to Mn-SOD deficiency might induce the degradation of the SDH protein. In the heart mitochondria, the H/M-*Sod2*^−/−^ mice were previously shown to have significantly reduced ATP production. In fact, we revealed that the O_2_^•−^ formation in the mutant heart mitochondria was increased to 1.87-fold the level of the control mice. Recently, a flow cytometric analysis also demonstrated increased ROS production in isolated cardiomyocytes from H/M-*Sod2*^−/−^ mice [16]. In fibroblasts, Mn-SOD deficiency also markedly induced ROS generation, thus indicating that Mn-SOD depletion generally causes higher ROS levels in the cells (unpublished results).

## 3. EUK-8

**Figure 2:**
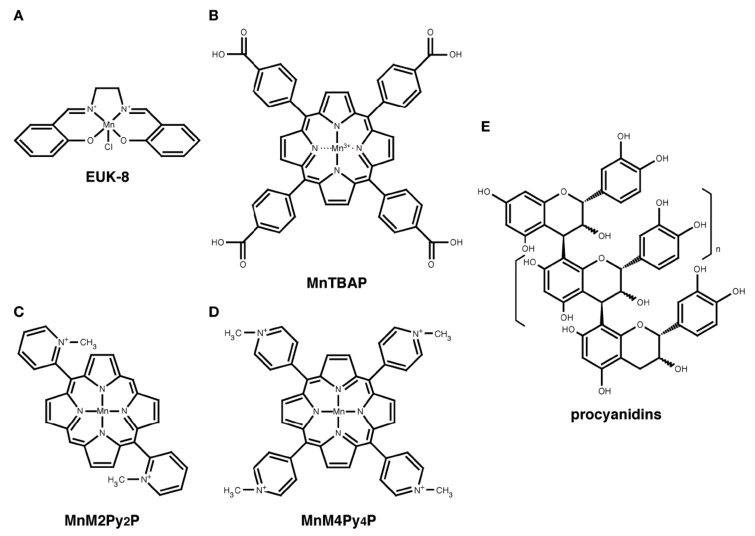
The chemical structures of the antioxidants described in this review. (**A**) EUK-8; (**B**) MnTBAP; (**C**) MnM2Py_2_P; (**D**) MnM2Py_2_P, and (**E**) procyanidins.

EUK-8 is a synthetic salen-manganese complex, which has both SOD and catalase activities (Figure 2A) [17,18]. EUK-8 is a potent SOD/catalase mimetic and antioxidant. EUK-8 can dismutate O_2_^•−^ to H_2_O_2_ and catalyze the breakdown of H_2_O_2_ to O_2_ and H_2_O. Some *in vivo* studies have shown that EUK-8 has protective effects in model organisms with ROS-induced diseases, such as heart mitochondrial dysfunction, aging, adrenergic hypertrophy, ischemia-reperfusion injury, and postischemic reperfusion arrhythmias [17,19,20,21,22]. Interestingly, EUK-8 was demonstrated to extend the lifespan of wild-type nematodes and systemic *Sod2* knockout mice [23,24]. These results suggest that EUK-8 scavenges the ROS generated in the cytoplasm or organelles, including the mitochondria.

We have previously reported the effect of EUK-8 on H/M-*Sod2*^−/−^ mice [25]. When EUK-8 was injected intraperitoneally (30 mg/kg/day) into the H/M-*Sod2*^−/−^ mice for four weeks, cardiac dilation and pump failure were significantly prevented [25]. The ROS generation in heart mitochondria was also reduced by EUK-8 treatment, suggesting that EUK-8 is a mitochondria-targeting antioxidant. Interestingly, EUK-8 therapy also significantly reversed the established DCM-like phenotypes in H/M-*Sod2*^−/−^ mice [25]. This was the first preclinical data indicating that EUK-8 is a useful new drug for the treatment of DCM. Furthermore, our findings revealed that the mimetic treatment normalized the molecular changes due to DCM, such as those in connexin43, telomerase reverse transcriptase (mTERT), telomeric repeat binding factor 2 (TRF2), phospho-AKT, insulin-like growth factor (IGF-1), endothelial nitric oxide synthase (eNOS), SIRT1, and FOXO3a [25,26]. These results suggest that the administration of antioxidants significantly restores cardiac contractility, as well as normalizing the molecular alterations in H/M-*Sod2*^−/−^ mice.

## 4. MnTBAP

MnTBAP is a metalloporphyrin that is widely used as an SOD/catalase mimetic worldwide (Figure 2B) [27]. We examined the effect of MnTBAP on H/M-*Sod2*^−/−^ mice [15]. Although the heart weight/body weight of H/M-*Sod2*^−/−^ mice was not rescued by the intraperitoneal administration of MnTBAP, the cardiac function of H/M-*Sod2*^−/−^ mice was significantly improved by MnTBAP treatment. MnTBAP treatment also recovered the daily running distances and the rotarod tasks in the H/M-*Sod2*^−/−^ mice [15]. These data suggested that MnTBAP treatment significantly rescued the impaired cardiac contractility, as well as the physical disabilities, of the H/M-*Sod2*^−/−^ mice [15].

## 5. Manganese Porphyrins

Manganese (Mn) porphyrins are water-soluble SOD/catalase mimic complex and have antioxidant activity under some conditions [28,29,30]. Recently, Hayakawa *et al.* reported that the Mn-porphyrins, 5,15-bis(2-methylpyridyl)porphinato manganese (MnM2Py_2_P), and 5,10,15,20-tetrakis(4-methyl-pyridyl)porphinato manganese (MnM4Py_4_P) (Figure 2C,D) [30,31], decreased the oxidative stress and heart weight of H/M-*Sod2*^−/−^ mice [32]. MnM2Py_2_P could suppress the pathology of DCM more effectively than MnM4Py_4_P, and is expected to be an effective drug against DCM. Taken together with the data about EUK-8 and MnTBAP, antioxidants with SOD activity may protect hearts from mitochondrial ROS in bioavailability- and drug metabolism-dependent manners, rather than by their antioxidative activity, *in vivo*.

## 6. Procyanidins

Polyphenols are a structural class of mainly natural, but also synthetic, organic chemicals characterized by the presence of large and multiple phenol structural units. Polyphenols comprise several groups of compounds, such as anthocyanins, flavonols, and phenolic acids, and are accumulated in plants as well as fruits [33]. Resveratrol is a well-known polyphenol, which can extend the lifespan of yeast, worms, flies, and mice [34,35,36]. Several studies have reported that dietary polyphenols from vegetables and fruits prevent oxidative stress *in vivo* [37,38].

The apple polyphenols (AP) have various physiological functions, such as anti-oxidant, anti-tumor, anti-allergy, and anti-obesity effects [16,39,40,41]. The major polyphenols contained in apple are procyanidins (PC), which are composed of (–)-epicatechins and (+)-catechins (Figure 2E) [42]. PC are also contained in other fruits, such as grape seeds and blueberries [43].

Previously, we tested the effect of PC on the lifespan of *Caenorhabditis elegans* [44]. Treatment with AP and PC could significantly extend the mean lifespan of wild-type N2 and *fem-1* mutant worms. However, PC could not extend the longevity of *mev-1* and *sir-2.1* mutant worms. The *mev-1* strain is a short-lived mutant that suffers from excessive oxidative stress [45]. This suggested that PC extends the lifespan in *C. elegans* in an antioxidant-independent manner, although PC showed a strong antioxidant activity *in vitro* [46]. The *sir-2.1* worm is a mutant that lacks histone deacetylase SIR-2 activity [35]. The findings in this strain indicated that PC prolongs the lifespan of worms in a *sir-2*-dependent manner. In an *in vitro* HDAC assay, PC failed to activate SIRT1. It has been reported that the up regulation of the *sir-2* gene extends the survival in worms [47]. Therefore, it seems possible that PC might regulate *sir-2* expression. Since NAD is essential for SIR-2 activity, it is possible that PC controls NAD metabolism. Wilson *et al.* reported that PC from blueberries extended the lifespan of worms in a *sir-2-*independent manner [48]. Therefore, it is possible that the PC from apples and blueberries extends the lifespan of worms by different mechanisms.

Recently, we examined the effect of AP on H/M-*Sod2*^−/−^ mice [16]. When AP were administered to H/M-*Sod2*^−/−^ mice in drinking water containing 0.1% AP, the AP significantly extended the lifespan of the H/M-*Sod2*^−/−^ mice, and was associated with an attenuation of the enlarged heart from 22 weeks to 29 weeks of age. AP treatment also mitigated the accumulation of the plasma creatine phosphokinase (CPK) level, histopathology, and fibrosis in the heart of H/M-*Sod2*^−/−^ mice. The biochemical analyses using ROS-reacting fluorescent reagents revealed that AP supplementation reduced the ROS production in the cardiomyocytes of mutant hearts *in vivo*. *In vitro* experiments also demonstrated that AP and PC treatment significantly decreased the ROS production in fibroblasts isolated from systemic conditional *Sod2*^−/−^ mice, indicating that AP and PC improved the DCM-like phenotypes of H/M-*Sod2*^−/−^ mice by an antioxidative action [16].

## 7. Antioxidants for Human DCM

Though we have described about the effects of antioxidants on the DCM model mice, some antioxidative drugs have been already used to human DCM patients. Coenzyme Q_10_ (CoQ_10_) is a small redox active lipid, which is containing in all membrane and is an electron transporter in the respiratory chain of mitochondria [49,50,51]. Manzoli *et al.* reported that the beneficial effect of CoQ_10_ on DCM patients [52]. The administration of CoQ_10_ could improve ejection fraction, telediastolic volume, and reduced CoQ_10_ plasmatic level of DCM patients [52]. Carvedilol, one of β-adrenoceptor blockers has an antioxidant activity and is used to therapy for human DCM [53,54,55]. Carvedilol therapy reduced the overall mortality rate and the risk of hospitalization [54]. These clinical data suggest that antioxidants are useful to the therapy for DCM patients.

## 8. Effects of EUK-8 on Muscle

**Figure 3:**
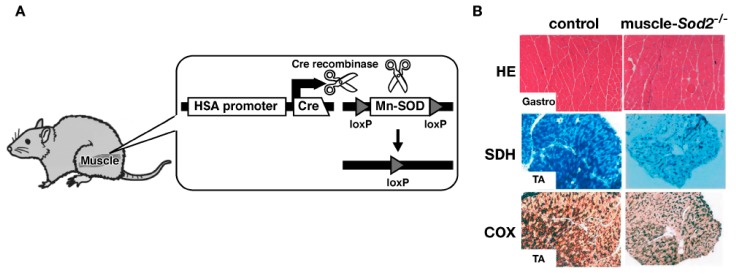
Muscle-specific Mn-SOD-deficient mice. (**A**) Cre recombinase is expressed under the control of a skeletal muscle-specific human skeletal actin (HSA) promoter. Cre recombinase recombines the loxP sites selectively to generate muscle-specific Mn-SOD-deficient mice; (**B**) Sections of gastrocnemius (Gastro) and tibialis anterior (TA) muscles from control mice (left panels) and muscle-*Sod2*^−/−^ mice (right panels). Examples of HE staining (top panels) and enzymatic staining for succinate dehydrogenase (SDH, middle panels) and cytochrome c oxidase activity (COX, bottom panels) are shown.

We have reported the effect of EUK-8 on muscle specific *Sod2* knockout (muscle-*Sod2*^−/−^) mice [56]. The muscle-*Sod2*^−/−^ mice express Cre recombinase under the control of the human skeletal actin promoter (Figure 3A) [57]. Although muscle-*Sod2*^−/−^ mice showed the loss of SOD2 in their skeletal muscle, there was no difference in their outer appearance, body size, or food intake compared with control mice. Interestingly, muscle-*Sod2*^−/−^ mice showed centronuclear muscle with increased serum creatine phosphokinase activity, which is a typical hallmark of regenerative muscle, in spite of the fact that they exhibited no loss of muscle mass (Figure 3B) [56]. The biochemical analyses revealed significantly increased oxidative DNA damage, reduced respiratory enzyme activities, including NADH dehydrogenase (Complex I) and SDH (Complex II), and a reduced ATP level in the skeletal muscles of muscle-*Sod2*^−/−^ mice (Figure 3B) [56]. Intriguingly, the muscle-*Sod2*^−/−^ mice showed normal spontaneous activity during free movement in a cage, while they exhibited severe exercise disturbance on a forced running task using treadmill. When a single dose of EUK-8 was injected intraperitoneally into muscle-*Sod2*^−/−^ mice, the decreased ATP content in the skeletal muscle and running disturbance in the mutant mice were markedly attenuated at 24 hours after the injection [56]. These data suggest that EUK-8 is useful for protecting the mitochondrial function in skeletal muscle from the ROS generated by the mitochondria *in vivo*, and demonstrate that the muscle-*Sod2*^−/−^ mouse is a beneficial model for investigate the cellular mechanism(s) of muscle fatigue.

## 9. Conclusions 

In this review, we introduced the tissue-specific Mn-SOD knockout mice and summarized our recent studies on antioxidants using these mice. The H/M-*Sod2*^−/−^ and muscle-*Sod2*^−/−^ mice are useful mouse models for DCM and muscle fatigue, respectively. Antioxidants such as EUK-8, MnTBAP, Mn-porphyrins, and procyanidins efficiently improved the phenotypes of H/M-*Sod2*^−/−^ mice and/or muscle-*Sod2*^−/−^ mice. These data suggest that these antioxidative compounds are beneficial for the treatment and/or prevention of some diseases caused by ROS-mediated mitochondrial dysfunction.
